# Biomechanical and Biological Multidisciplinary Strategies in the Orthodontic Treatment of Patients with Periodontal Diseases: A Review of the Literature

**DOI:** 10.3390/bioengineering12010049

**Published:** 2025-01-09

**Authors:** Gaia Viglianisi, Alessandro Polizzi, Teresa Lombardi, Mariacristina Amato, Cristina Grippaudo, Gaetano Isola

**Affiliations:** 1 Department of General Surgery and Surgical-Medical Specialties, School of Dentistry, University of Catania, 95124 Catania, Italy; gaia.viglianisi@gmail.com (G.V.); alessandro.polizzi@phd.unict.it (A.P.); amato.mariacristina@hotmail.it (M.A.); gaetano.isola@unict.it (G.I.); 2 Department of Health Sciences, University Magna Græcia, 88100 Catanzaro, Italy; drteresalombardi@libero.it; 3 UOC di Clinica Odontoiatrica, Dipartimento di Neuroscienze, Organi di Senso e Torace, Fondazione Policlinico Universitario A. Gemelli IRCCS, 00168 Rome, Italy; 4 Dipartimento Universitario Testa Collo ed Organi di Senso, Università Cattolica del Sacro Cuore, 00168 Rome, Italy

**Keywords:** orthodontics, biomechanics, periodontitis, root resorption, gingival recession

## Abstract

Orthodontic treatment aims to correct malocclusions and ensure the overall health and stability of the periodontium. The relationship between orthodontic therapy and periodontal health is intricate and multifaceted, and a comprehensive approach is often required to achieve optimal outcomes. Firstly, this article delves into the impact of orthodontic mechanics on periodontal tissues, emphasizing the importance of minimizing iatrogenic effects such as root resorption and gingival recession. Understanding the biomechanical principles allows for the development of treatment plans that mitigate these risks while achieving the desired tooth movement. Effective communication and coordinated treatment protocols are essential for managing periodontal issues before, during, and after orthodontic intervention. To optimize outcomes, periodontal considerations such as gingival biotype, attachment levels, and bone support must be integrated into treatment planning. Additionally, adjunctive periodontal therapies such as selective alveolar decortication and regenerative procedures are explored as valuable tools to enhance periodontal support and optimize treatment outcomes. This narrative review explores strategies to attain periodontal goals in orthodontic patients, thereby facilitating successful treatment. Furthermore, the review examines the role of interdisciplinary collaborations between orthodontists and periodontists. In conclusion, achieving periodontal goals in orthodontic patients requires a comprehensive approach that addresses biomechanical principles, interdisciplinary collaboration, patient education, and adjunctive periodontal therapies. By integrating periodontal considerations into orthodontic treatment planning and execution, clinicians can ensure straight teeth and a healthy and stable periodontium, ultimately leading to successful treatment outcomes and long-term oral health.

## 1. Introduction

Periodontitis is a multifactorial chronic disease caused by a dysbiosis gingival biofilm and characterized by the progressive destruction of the tooth-supporting apparatus that can lead, if not properly treated, in tooth loss [1]. Periodontitis, especially its mild and moderate forms, is highly prevalent in adult-aged populations all over the world, with prevalence rates of around 50%, while its severe form affects around 10% of the adult population worldwide [2,3]. Alterations in clinical attachment level (CAL) are the main sign of the disease and allow for the diagnosis of periodontitis [4]. Even though oral microbial and genetic predisposition are the two main factors in the development of periodontitis, others can contribute to an increase in the degree of its pathology, such as diabetes, cardiovascular conditions, smoking, and occlusal trauma [5]. Without periodontal therapy, the progression of inflammation causes an increase in periodontal attachment and bone loss, which can lead to tooth mobility, rotation, migration, and super-eruption [6]. Occlusal trauma is another complication that can occur due to the progression of periodontitis and also contributes to the development of the disease. Indeed, altered occlusion leads to constant trauma in the periodontium, increasing bone and attachment loss, involving specific mediators such as microRNAs [7,8]. These dental alterations need to be treated via orthodontic correction. This is why orthodontic specialists are included in the multidisciplinary approach to managing periodontopathic patients [6].

In the dental field, orthodontics is focused on diagnosing and treating malocclusions using different orthodontic devices, including braces and clear aligners. These devices release a force on the tooth, aiming to achieve alignment. Due to the increase in requests for orthodontic therapy in adults, interest in the interplay between periodontitis and orthodontics has increased in dental research. Orthodontic movement has implications for the periodontium. In the case of a compromised periodontium, the advised application of orthodontic force changes compared to that advised for individuals without periodontitis. The work of a multidisciplinary team of specialists is necessary for the treatment of periodontitis. The multidisciplinary team should include an orthodontic specialist to treat the functional and esthetic sequalae that periodontitis may induce. Moreover, the role of orthodontists is to resolve crowding, which hinders domiciliary oral hygiene procedures. The aim of this narrative review is to explore strategies that could help to achieve periodontal goals in orthodontic patients, thereby facilitating successful treatment. Furthermore, this review examines the role of interdisciplinary collaboration between orthodontists and periodontists.

## 2. The Interplay Between Orthodontic Therapy and Periodontal Health

### 2.1. Orthodontic Mechanics and Periodontal Tissues

The biology behind orthodontic tooth movement consists of several phases and processes. Normally when a force is applied on a tooth, a physiological process begins in the periodontal tissues, including the formation of two zones: a pressure zone and a tension zone. This physiological mechanism is called ‘the pression–tension theory’. In the pressure zone, the initial tooth movement impacts the small amount of gingival space due to the leakage of crevicular fluids. Additionally, in this region, the blood vessels are blocked, which causes the hyalinization process to begin. This process lasts from six to eight days. Until hyalinization tissue is present, tooth movement is blocked, and after this period, the hyalinization zone is gradually removed and dental movement begins [9,10]. In a compromised periodontium, the development of the hyalinized zone is harmful due to inflammation and the periodontium cannot regenerate in the tension zone, while only bone resorption occurs in the pressure zone. Instead, in a healthy periodontium, the hyalinized zone is transformed into new bone before its subsequent removal, allowing for tooth movement. This is one of the reasons why inflammation caused by periodontitis has to be arrested before an orthodontic movement.

**Figure 1:**
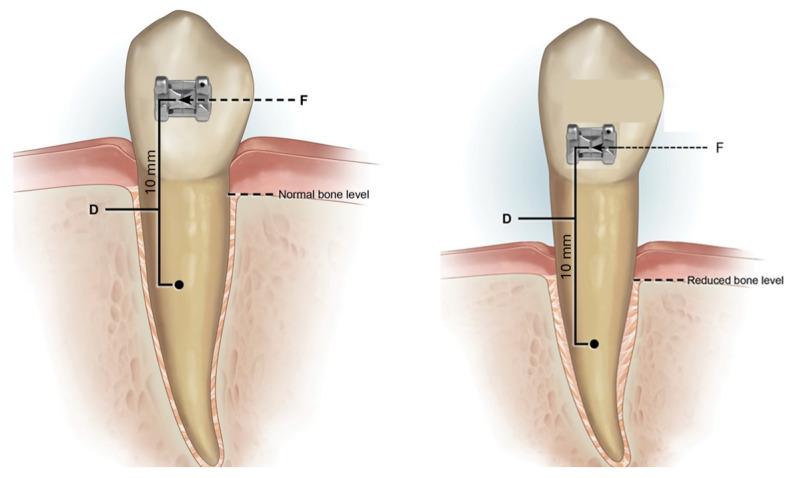
Biomechanically, the brackets should be located 10 mm from the center of resistance. In cases where a periodontal patient has a reduced bone level, the center of resistance of the tooth changes. The brackets should be positioned as cervically as possible to maintain this distance based on the residual bone level.

Different aspects of orthodontic biomechanics planning must be considered in a compromised periodontium. In a healthy periodontium, the center of resistance is located in the middle third of the root. In cases of alveolar bone resorption, the center of resistance is located apically. This means that there is high possibility that tipping occurs more likely than translation movements under force [11,12]. Due to bone loss, the moment/force relation also changes. In a healthy periodontium, the mean distance between the center of resistance and the bracket’s position is about 10 mm [13]. In a compromised periodontium, the brackets should be placed as cervically as possible based on the bone level to maintain this distance. Additionally, the more distance there is between the center of resistance and the point of application of the force, the greater the tooth rotation that will be obtained [14,15,16] (Figure 1).

Biomechanically, in this case, to achieve bodily movement, devices such as power arms and sectionals are preferable to continuous-arc wires because they allow for the direct application of force to the center of resistance of the teeth [17]. In cases of alveolar bone loss, the applied forces will be located apically. Various studies have shown this method of the dissipation of force to increase the risk of tooth resorption [18,19,20]. Secondly, the anchorage must be well-planned to counteract unwanted tooth movements, especially in cases of concomitant systemic diseases [21]. In periodontal patients, it may be risky to use teeth as a form of anchorage, while in a partially edentulous mouth, it may be difficult or impossible to find the necessary supports for anchorage [22]. Posterior teeth are commonly used as a means of anchorage, but they require a sufficient periodontium attachment to perform this role. Extrusive movement is a common side effect in teeth with reduced periodontium when used as an anchorage [17,22,23,24]. Different orthodontic devices, including a trans-palatal bar [16,23], a Nance button, and a bite block, may reduce the extrusive side effects that occur during other tooth movements [17,22,23,24]. Usually, orthodontic therapy has to be preceded by periodontal therapy but there are exceptions in which the orthodontic therapy is carried out before [25]. Sometimes, a candidate tooth for extraction can be used as a means of anchorage for the teeth alignment. In this way, the crowding may be resolved using the unsavable tooth as an anchorage, and at the end of the alignment, the tooth may be extracted [26]. If the posterior region is edentulous, temporary anchorage devices (TADS) and dental implants can be used as a stable anchorage to help orthodontic movements [22,27]. However, the use of many tooth movement support devices may constitute an obstacle to daily oral hygiene, requiring greater patient compliance. Concerning the biomechanical and physiological aspects of this treatment, the intensity of the force should be as light as possible due to the reduced bone support [14,28]. Knowledge of these concepts is fundamental when planning a personalized orthodontic treatment.

Moreover, it is common to find over-erupted teeth in patients with periodontitis. In this case, the intrusion is a mandatory orthodontic movement. In a study conducted by Ericsson et al. [29], intrusion caused the migration of the supra-gingival plaque in the sub-gingival side, allowing for the development of infra-bony pockets. Despite this, the intrusion movement may only be performed if oral plaque is controlled and the orthodontic forces are well-calibrated and have a low magnitude [27,30]. In fact, different histological studies showed that intrusion positively impacted the compromised periodontium, increasing the cellular density, mitosis, and cementum formation, and reducing the periodontal ligament’s width [31,32,33]. This is probably related to the fact that the intrusion stretching force may create a barrier, causing the epithelial cells to growth apically [34].

Furthermore, in different studies it was shown that, in patients with infra-bony defects related to periodontitis, the presence of a mechanical trauma may induce the evolution and progression of the periodontal attachment loss [35,36]. This evidence underlines the importance of properly treating infra-bony defects before beginning the orthodontic movement due to the high possibility of worsening the periodontal attachment loss [37].

### 2.2. Iatrogenic Effects: Root Resorption and Gingival Recession

Orthodontic movement may expose the teeth to different adverse effects, including root resorption and gingival recession.

Orthodontic root resorption is defined as an aseptic inflammatory process that induces a modification of the root cementum or dentin [38] via osteoclast and cemetoclast activity [39]. The reason root resorption occurs is still unknown, but its pathogenesis appears to be multifactorial [40]. The duration of the orthodontic treatment is a risk factor for root resorption. There are various possible reasons that treatment duration may be extended, including inadequate patient collaboration, complexity in tooth movements, and changes in the treatment plan [41,42,43,44]. The type of orthodontic movement, the magnitude and the intensity of the force, occlusal trauma, and periodontitis are other risk factors related to root resorption [45]. In a study conducted by Shen et al. [19], different studies of orthodontic treatments conducted in patients with periodontitis were analyzed. These studies’ results highlighted that tooth resorption risk is higher in cases of intrusion and during the bodily movement of lower and upper incisors to resolve sequelae related to periodontitis. Similar conclusions were reached in the study by Choi et al. [14]. Therefore, a low magnitude force, a careful evaluation of dental anatomy, and bone support are fundamental to achieve orthodontic movement with fewer side effects.

Gingival recession is defined as the migration of the gingival tissues apically to the cervical enamel junction (CEJ) position. During gingival migration, the root is exposed to an oral environment, leading to clinical problems such as cervical cavities, abrasion, hypersensitivity, and unesthetic issues [46,47]. The prevalence of gingival recession ranges from 5% to 12% when orthodontic therapy is completed. Gingival recession can be present in all types of teeth, but it was noted that mandibular incisors are more prone to recession [48]. Thin gingival tissues, age, tooth movement, tooth morphology, fenestration and dehiscence, thin alveolar bone, and patient susceptivity are several risk factors for gingival recession [49,50]. Furthermore, gingival recession may also be a consequence of periodontitis or an ectopic tooth position [51], particularly in patients with thin gingival biotypes. This aspect must be considered when orthodontic treatment is planned because orthodontic movements in patients with thin gingival biotypes can cause gingival recession. For this reason, if gingival recessions are present before the beginning of orthodontic treatment, mucogingival surgery to ensure root coverage and incremental gingival treatments could be planned. Conversely, if gingival recession occurs as an orthodontic sequel, mucogingival surgery may be performed after the orthodontic therapy [52,53]. Gingival recession may appear after finishing orthodontic treatment or could be exacerbated during orthodontic tooth movement.

## 3. Interdisciplinary Collaboration: Orthodontist–Periodontist Partnerships

### 3.1. Communication and Treatment Coordination

**Figure 2:**
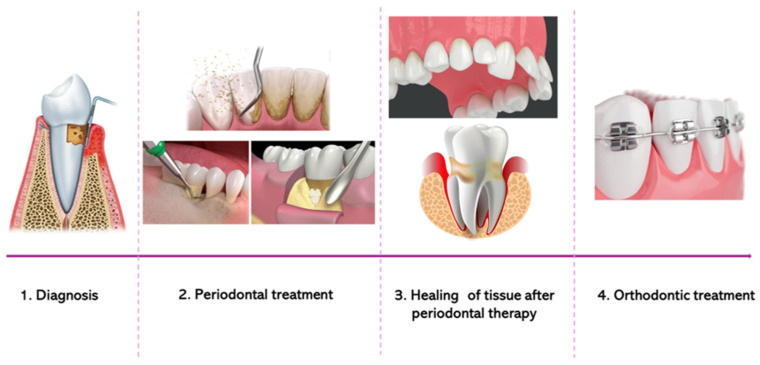
Graphic representation of the different phases to include in cases in which orthodontic therapy should be carried out for a periodontopathic patient.

A pyramid treatment planning scheme has been proposed in different papers, requiring cooperation between periodontologists and orthodontists. Different phases should be included in the treatment planning when orthodontic therapy is required in patients with periodontitis [7,11,19,22,23,27,28,54] (Figure 2).

After periodontitis is diagnosed, professional periodontal treatment is started. The main objectives are to arrest inflammation, establish good domiciliary oral hygiene, reduce or eliminate periodontal pockets, and increase the attachment level. All of these objectives require time, and orthodontic treatment cannot be started before the periodontal tissue has healed. The healing time may vary based on the periodontal procedures used [18,55,56,57]. If necessary, regenerative, conservative, mucogingival, and osteoresective surgery requires a prolonged healing time compared to non-surgical periodontal therapy [28]. Firstly, periodontal inflammation and resorption have to be arrested and controlled. As suggested by the international guidelines, the treatment of periodontitis is based on a primary active therapy or non-surgical therapy. During that phase, the patient is taught to follow correct domiciliary plaque control measures and motivated to remove risks factors like tobacco. Moreover, using ultrasonic instruments, the hygienist performs professional hygiene procedures to remove the subgingival bacterial biofilm. After non-surgical therapy, if there are still periodontal pockets with a depth of ≥ 6 mm, periodontal surgery may be required [4,57]. After surgical treatment, the orthodontic treatment can begin once the periodontal tissues heal. Deciding on the best time to begin orthodontic movement after non-surgical or surgical periodontal therapy is sometimes difficult. Prato et al. [58] created an algorithm to help clinicians select the best time to begin this therapy. The authors suggested performing orthodontic movement 3–6 months after non-surgical therapy, 6–9 months after an open-flap debridement procedure, and after 1 year in the case of regenerative surgery. These suggested timings were based on scientific evidence from the literature and the biology of wound-healing [58]. According to the guidelines, it is not necessary to wait a longer time after regenerative procedures to start orthodontic treatment because there are no significant differences in periodontal parameters that indicate that it is better to start after a long healing period [54]. During orthodontic treatment, the multidisciplinary team has to monitor and measure the periodontal clinical parameters which, as indicated by the international guidelines, should be as follows: plaque index < 20%, bleeding on probing < 20%, and periodontal probing depth < 5 mm [4,58,59]. These parameters may increase during orthodontic treatment, indicating a new exacerbation of periodontitis. In this case, orthodontic movement must be stopped and active periodontal therapy must be started again. Only when periodontal parameters are restored can the tooth movement be continued [57].

At present, in the orthodontic field, fixed multi-brackets, lingual brackets, a single wire, and clear aligners can be used to correct malocclusions. Depending on the malocclusion and the orthodontic movement needed, clinicians may propose different treatment strategies. In the case of periodontal patients, if there are several possible ways to correct the malocclusion, the preference is to use the shortest treatment and the option that will least hinder oral hygiene [60]. In periodontal patients, fixed orthodontic appliances are preferable, even if the recent introduction of clear aligners which exert a light force has changed the general direction of treatment [61,62].

Clear aligners are removable orthodontic devices that have recently been introduced in orthodontics. To allow for orthodontic movement while using the device, clinicians apply little attachments (made of composite resin) to the tooth’s surface. Clear aligners are always customized to the patient to allow for the desired tooth movement. One advantage of this technique is the possibility of projecting each tooth movement prior to the fabrication of the aligners [63]. Therefore, in patients with periodontitis, these orthodontic systems may present some advantages, including the ability to adjust the amount of force imparted by the aligners and lengthen the time required to achieve movement. If properly projected, personalized clear aligners may place less stress on periodontal tissues. Another important advantage of aligners is their removability, which allows for better cleaning. However, in patients with advanced periodontitis, tooth mobility and active inflammation are contraindications to using aligners as the progression of the disease could be aggravated due to the insertion and removal maneuvers [64].

At the end of orthodontic therapy, fixed or removable retainers may be used to maintain the occlusion achieved. According to the guidelines, retainers are recommended in periodontal patients who received orthodontic treatment. Fixed retainers are preferred over removable ones. Long-term supportive periodontal care and orthodontic periodical check-ups should take place after the retainer is positioned. Check-ups during the retention period allow for the dentist to intercept the onset of unwanted tooth movements, which the forces exerted by the retainers could cause. These devices should be passive, but some cases they have been reported to exert active forces capable of causing unwanted tooth movement and gingival recession [65,66,67]. Additionally, these fixed devices make plaque removal difficult. This long-term supportive care should be personalized and based on the clinical oral patient’s characteristics [57].

### 3.2. Periodontal Considerations in Treatment Planning

**Figure 3:**
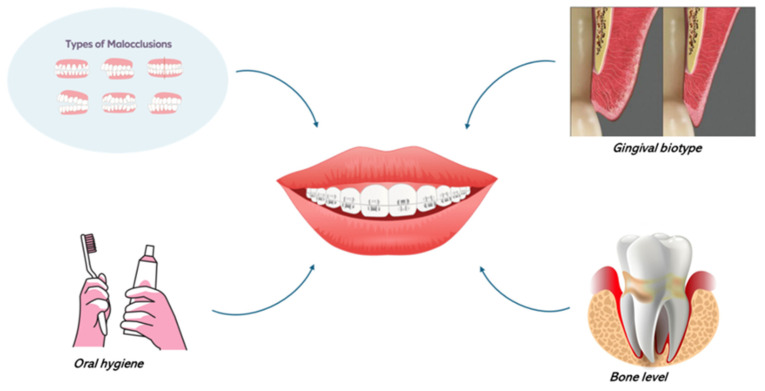
Graphic representation of the aspects that should be taken into account when orthodontic treatment is offered to patients with periodontitis.

Different factors should be evaluated when a combined periodontal–orthodontic treatment is planned in adults affected by periodontitis. These are the periodontal condition, malocclusion, periodontal bone loss, periodontal biotype, and oral hygiene level [68] (Figure 3).

As mentioned before, orthodontic therapy may be an option for patients affected by periodontitis with reduced but healthy periodontium. Conversely, in patients with active periodontitis, it is mandatory to start active periodontal therapy before orthodontic treatment. Performing orthodontic movements during active periodontitis can speed up alveolar bone loss [29,69,70]. Another aspect to take into consideration is the variability of the host’s periodontal inflammatory response. Periodontitis is a chronic inflammatory disease with variability in its manifestation, duration, and effects [71]. Therefore, monitoring periodontal parameters is mandatory throughout the duration of orthodontic treatment. Clinical periodontal indices worsening could exacerbate the disease at a specific subgingival site, which should be monitored.

Furthermore, the type of malocclusion being treated in a patient with periodontitis influences the choice of therapy. A tooth with a negative prognosis may be maintained as the point at which the force reaction can be discharged. The presence of crowding increases the possibility of gingival recession after alignment.

The remaining bone support is a fundamental aspect that an orthodontic specialist needs to know. Tooth movement is based on biomechanical factors. A reduction in bone support changes the center of resistance of the tooth. This implies that this tooth is more prone to tipping movements instead of a bodily movement [14,15].

Additionally, the intensity of the orthodontic force is dissipated when support is reduced compared to a healthy periodontium. Infra-bony defects can be present at the end of non-surgical therapy and it is only possible to induce orthodontic movement in these sites if inflammation is arrested. It is also possible to perform regenerative therapy before the beginning of the tooth movement.

Generally, during the orthodontic planning stage of a healthy patient’s case, the gingival biotype is taken into consideration because it is well-known that certain orthodontic movements could induce gingival recession. Evaluating gingival biotype in periodontal patients is fundamental for establishing a good treatment plan when orthodontic therapy is involved. In fact, if augmentation via oral surgery is necessary, this procedure should be performed before the beginning of the tooth movement [68,72].

Educating the oral hygiene patient is part of the non-surgical therapy and the goal is to teach and motivate the patient to follow a correct household plaque control strategy [73]. A patient with good oral hygiene is a compliant candidate that will follow the orthodontist’s advice during the therapy. Compliance is essential in both periodontal and orthodontic therapy.

All these aspects are important during orthodontic diagnosis and treatment planning, allowing for a personalized approach based on the periodontal patient’s characteristics.

## 4. Patient Education and Oral Hygiene Instruction

**Figure 4:**
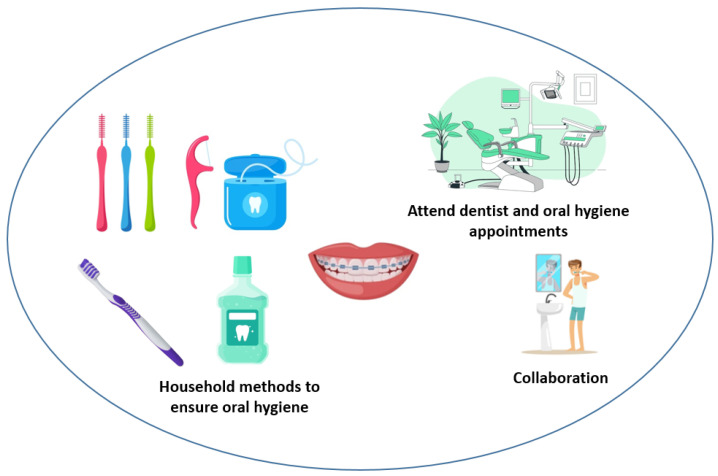
Illustration of the oral hygiene practices that patients with periodontitis must follow during and after orthodontic therapy.

Generally, one of the side effects of orthodontic devices is that they increase plaque accumulation. For this reason, oral hygiene control is necessary to avoid gingivitis, tooth cavities, decalcification, and the development of periodontitis triggers. The role of dental hygienists and orthodontists is to inform and educate patients on the proper use of oral domiciliary hygiene techniques [74,75,76]. Additionally, periodical oral hygiene control and professional sessions have to be planned during orthodontic treatment. These rules are important for healthy patients and even more so for individuals with periodontitis (Figure 4) because orthodontic force cannot be applied to teeth with active periodontitis [75,76]. Oral hygiene appointments should take place during and after orthodontic treatment to maintain periodontal health and reduce the accumulation of plaque on the orthodontic devices. The frequency of these oral hygiene appointments is determined based on collaboration, periodontal status, and patient history. Musilli et al. [60] suggested a 30 min appointment each month during orthodontic therapy in which professional oral hygiene advice and motivation are provided. If periodontal parameters are higher than 10% during one of these appointments, orthodontic therapy should be stopped, oral hygiene should be re-enforced, and motivation should be improved. The orthodontic therapy will be stopped until the periodontal parameters return to below the 10% score [60].

Orthodontic treatment has an influence on the organization and composition of oral microbiota [77], increasing the amount of periodontopathogenic bacteria. Therefore, orthodontic devices may be a risk factor for the re-activation of periodontitis in these patients [78].

## 5. Adjunctive Periodontal Therapy in Orthodontic Patients

### 5.1. Selective Alveolar Decortication

Orthodontic movement is more difficult in adult patients compared to younger individuals. In fact, bone turnover is faster in adolescents and periodontal status is healthier. Gingival recession and periodontitis are most commonly encountered in adults and adult patients have more tissue resistance and slower bone turnover than young patients. These factors have led to corticotomy procedures being used as an adjunctive tool in the orthodontic treatment of adult patients [79,80].

Corticotomy was first introduced in the orthodontic field in 1959 by Kole [81]. Corticotomy-assisted orthodontic treatment (CAOT) consists of the corticotomy procedure and the application of orthodontic force to the teeth immediately after surgery. CAOT has been shown to enhance different tooth movements, such as molar intrusion, the resolution of open bite, tooth anchorage, canine distalization, the alignment of crowing, and long-term tooth stability [82] (Table 1). Corticotomy is a surgical procedure that results in a reduction in the cortical bone of the alveolar bone without touching the medullar bone [83]. Corticotomy creates trauma in a specific zone of the alveolar bone, which results in the activation of bone turnover and healing. This phenomenon was seen to accelerate orthodontic movement, reducing the duration of orthodontic treatment [84]. Recently, bone grafting in the decorticate bone zone was introduced as a procedure in corticotomy-assisted orthodontic treatment. Despite the positive results that can be obtained using the CAOT, limitations are present. This procedure cannot be used for all types of malocclusion [85], especially in patients with several oral diseases [82] or endodontic lesions, or in those taking bisphosphonates and nonsteroid anti-inflammatory drugs [80] who are treated with radiotherapy. Additionally, complications may occur, including root resorption [86], gingival recession, interdental bone decrease, soft tissue reduction, and bone defects [87]. Correct planning of the surgical flap design could avoid some of these complications.

### 5.2. Periodontal Regenerative Procedure

Infra-bony defects may occur if periodontitis is not treated early. The guidelines recommend regenerative procedures when there are pockets with infra-bony defects deeper than or equal to 3 mm. Regenerative surgery is a surgical procedure that includes the removal of pathogenic bacterial biofilm and inflamed tissue inside of the pockets. After that, biomaterials are added to the defects to allow for the regeneration of hard and soft tissues. Different studies reported analogous effects if the orthodontic movement is started earlier or after a prolonged surgical procedure [61,62,88,89,90] (Table 1).

## 6. Limitations and Future Directions

The current analysis is limited due to its narrative review design (not a systematic review or a meta-analysis). For this reason, it cannot bring new knowledge to the topic. However, it provides a critical summary of orthodontic strategies used for the treatment of periodontal patients.

Modern dentistry is increasingly connected to the general well-being of the individual. The interdisciplinarity between orthodontics and periodontics that is required also broadens its impact.

First, the diagnostic aspects should be considered. The clinical aspects of malocclusion are derived from environmental and genetic risk factors. Many studies have been carried out on the role of environmental risk factors and the mechanics of orthodontic movement. The effect of orthodontic forces has been demonstrated using the rules of physics and mechanics. The results of this form the basis of current orthodontic techniques that use fixed or removable devices to determine the desired tooth movement [91]. Scientific research has also contributed to investigations of the biological aspects of tooth movement. In this field, theories and knowledge have changed with progress in the means and methods of investigation [92]. The next step is understanding the role of the stem cells, growth factors, and signaling molecules underlying the changes that determine tooth eruption and the remodeling or destruction of the alveolar bone [93,94]. The same phenomena form the basis of bone resorption following periodontal disease. From the perspective of personalized medicine, the research aims to investigate the predisposing factors for the resorption of alveolar bone and dental roots, which must be considered when planning the chosen therapy [95].

Certainly, this multidisciplinary path offers interesting stimuli and perspectives that will lead to ortho-periodontal patients being treated in an increasingly accurate and safe manner.

## 7. Conclusions

Untreated periodontitis involves several sequelae that should be corrected to improve function and esthetics. Their treatment requires a multidisciplinary team consisting of periodontists, orthodontists, prosthetists, hygienists, dental implantologists, and endodontists. Coordination and collaboration between these dental specialties are fundamental at every stage, from diagnosis to treatment planning. In particular, interaction between periodontists and orthodontists is essential to orthodontic treatment in adults. In patients with a reduced periodontium, planned biomechanical procedures are used to achieve correct tooth movement changes and the orthodontists must use a specific orthodontic strategy that differs from that used for healthy patients. The alignment that is obtained must be maintained and patients should be followed-up for a long time. During and after orthodontic therapy, dental plaque control is mandatory to cleanly maintain the orthodontic devices used and avoid the accumulation of a pathogenic subgingival biofilm that could enhance periodontal inflammation. Frequent check-ups and the motivation of patients by clinicians allow them to maintain the patient’s collaboration and the obtained results.

## Figures and Tables

**Table 1:** Periodontal surgical procedures that can be performed in addition to orthodontic therapy in patients with periodontitis.

Adjunctive Periodontal Therapy in Orthodontics		
Goals		
Corticotomy	Accelerate orthodontic movement in the area in which was a corticotomy procedure was performed.	[77]
Regenerative	Regenerate soft and hard periodontal tissue to allow for the closure of the periodontal pocket and reduction in bone defects. Better anchorage during the orthodontic movement is another benefit obtained from this surgical procedure.	[58,59,83,84,85]
